# Impact of Early Versus Late Treatment with Botulinum Toxin A on Goal Attainment in Post-Stroke Spasticity: A Retrospective Cohort Study

**DOI:** 10.3390/toxins18020068

**Published:** 2026-01-27

**Authors:** Atul Patel, Jinming Zhang, Simon Page, Sarah Harding, Mathieu Beneteau, Colin Navickas, Alberto Esquenazi

**Affiliations:** 1 Kansas City Bone & Joint Clinic, Overland Park, KS 66211, USA; apatel@kcbj.com; 2 Ipsen, Cambridge, MA 02142, USA; jinming.zhang@ipsen.com; 3 Ipsen, London W2 1AF, UK; simon.page@ipsen.com (S.P.); sarah.harding@ipsen.com (S.H.); 4 Ipsen, 75015 Paris, France; mathieu.beneteau.ext@ipsen.com; 5 Genesis Research Group, Hoboken, NJ 07030, USA; cpnavickas@gmail.com; 6 Jefferson Moss-Magee Rehabilitation, Elkins Park, PA 19027, USA

**Keywords:** botulinum toxins, type A, muscle spasticity, stroke rehabilitation, treatment outcome

## Abstract

This study evaluated the effect of time of botulinum toxin A (BoNT-A) treatment on clinical outcomes in adults with post-stroke spasticity (PSS). Individual data were pooled from five studies. Eligible patients received ≥1 BoNT-A injection(s) for PSS and had goal attainment scaling (GAS) scores measured at baseline and 12 weeks. Patients were grouped according to time of treatment post-stroke: early (<1 year) or late (≥1 year). The primary endpoint was the total GAS (GAS-T) score change from baseline to 12 weeks. Secondary outcomes included the proportion of patients with a GAS-T score ≥ 50. Overall, 968 patients were included (166 early and 802 late). Median time post-stroke to BoNT-A treatment was 0.5 (early) versus 5.4 (late) years. Mean (standard deviation [SD]) baseline GAS scores were similar between cohorts (early: 36.9 [3.5]; late: 36.9 [3.6]). The mean (SD) change in the GAS-T score from baseline to 12 weeks was greater in the early versus late cohort (15.7 [8.9] vs. 13.1 [8.9]; *p* < 0.001). More patients in the early versus late cohort had a GAS-T score ≥ 50 (63.9% vs. 47.4%; *p* < 0.001) at 12 weeks. No new safety concerns were reported. Early treatment of PSS with BoNT-A has a positive impact on patients’ ability to achieve treatment goals. **Plain Language Summary:** After a stroke, people can experience muscle stiffness in their limbs, called post-stroke spasticity (PSS), which can lead to pain and make movement difficult. Treatment can include botulinum toxin A (BoNT-A) injections given directly into affected muscles. The aim of our study was to assess whether giving BoNT-A within a year after experiencing a stroke was more effective in treating PSS than delaying treatment. We combined data from 968 patients across five different studies. Most people (802 patients) received BoNT-A treatment 1 year or more after their stroke (late treatment group), while 166 people received treatment within a year of their stroke (early treatment group). In the studies, patients set treatment goals with their physician, for example being able to hold an object or walk a certain distance. After treatment, the extent to which each goal was achieved was assessed and scored based on whether the result was less than expected, as expected, or better than expected by the patient and physician. The scores from the two treatment groups were compared. People in the early treatment group did better in achieving their treatment goals compared with those in the late treatment group. We also looked at any side effects patients experienced. No unexpected side effects were reported. BoNT-A treatment of PSS can help patients achieve their treatment goals, and patients treated early (within 1 year after stroke) may do better than those treated later. This information may help in rehabilitation planning for stroke patients.

## 1. Introduction

Spasticity is a motor disorder characterized by a velocity-dependent increase in muscle tone that results in the loss of mobility and may produce pain due to muscle spasms. It is a classic clinical manifestation of an upper motor neuron lesion and involves damage to motor pathways in the brain or spinal cord [1,2]. Spasticity develops in approximately 25% of patients after stroke and up to 40% of patients with paresis after stroke [3]. The incidence of spasticity following stroke with paresis varies depending on the time period that has elapsed since stroke; it has been reported to be 36% within 1 month, 35% at 1–3 months, 42% at 3–6 months, and 45% beyond 6 months [3]. The variable prevalence of post-stroke spasticity (PSS) during the first year after onset makes consistent, timely diagnosis and optimal management challenging.

Early treatment of PSS is desirable to reduce complications such as pain and contracture, and to improve functioning [4]. Optimum use of the available therapeutic options for PSS requires early diagnosis and initiation of indicated interventions [1]; this can support physiotherapy and facilitate early rehabilitation, with benefits for both the patient and the healthcare system. Botulinum toxin A (BoNT-A) is a recommended first-line pharmacological treatment option for spasticity [1,5]; however, BoNT-A injections are not often initiated until muscle overactivity has been demonstrated, meaning that treatment is often delayed [6]. Consequently, studies evaluating the impact of early PSS treatment on treatment outcomes are limited [7,8]. Data from previous studies have indicated that there may be an advantage to early treatment, and that this advantage may be due to a neuromodulatory effect; however, studies have largely focused on reducing muscle tone, using measures such as the Modified Ashworth Scale (MAS), and improving passive function, with less emphasis on functional endpoints [9,10]. A recent secondary analysis of data suggested that early BoNT-A administration (within 3 months of stroke onset), particularly when combined with targeted neurorehabilitation, may be associated with enhanced motor recovery and additional functional gains [11]. However, the potential association of early BoNT-A treatment with improved goal attainment has not been established. Improvements in goal attainment scaling (GAS), a patient-centered functional endpoint, can capture clinically relevant improvements in daily life and may be more meaningful to patients than endpoints focusing on muscle tone alone.

By leveraging data pooled from five previously conducted prospective studies, this retrospective cohort study evaluated the impact of the timing of BoNT-A treatment for spasticity following a stroke on treatment outcomes, including the impact on patient-centered functional endpoints measured using GAS and the impact on muscle tone measured using MAS.

## 2. Results

### 2.1. Patient Population and Characteristics

**Figure 1:**
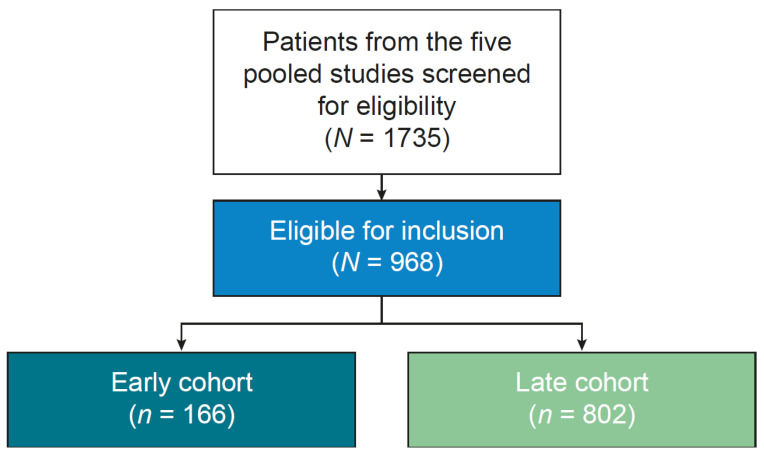
Study population. The early cohort consisted of patients who had BoNT-A treatment <1 year post-stroke. The late cohort consisted of patients who had BoNT-A treatment ≥1 year post-stroke. BoNT-A, botulinum toxin A.

Of the 1735 patients from the five pooled studies who were screened for eligibility, 968 patients were included in the analysis (Figure 1), of whom 58.4% were men and 38.0% were aged ≥ 60 years. Most patients had upper limb spasticity (ULS) only (81.9%), and the mean time from stroke to BoNT-A treatment was 6.7 years.

The study sample comprised 166 patients in the early treatment cohort and 802 patients in the late treatment cohort (Figure 1). Patient characteristics (age and location of spasticity) were generally balanced between the two cohorts, although there was a greater proportion of men in the early versus late treatment cohort (65.7% vs. 56.9%). The median time between stroke onset and BoNT-A treatment was 0.5 years in the early treatment cohort compared with 5.4 years in the late treatment cohort. As anticipated, most patients in the late treatment cohort (77.2%) had received previous BoNT-A injections compared with only 28.9% of patients in the early treatment cohort (*p* < 0.001). Notably, there was no significant difference in mean GAS score at baseline between the two cohorts. The demographic and baseline characteristics of the study population are presented in Table 1.

### 2.2. Primary Endpoint

**Figure 2:**
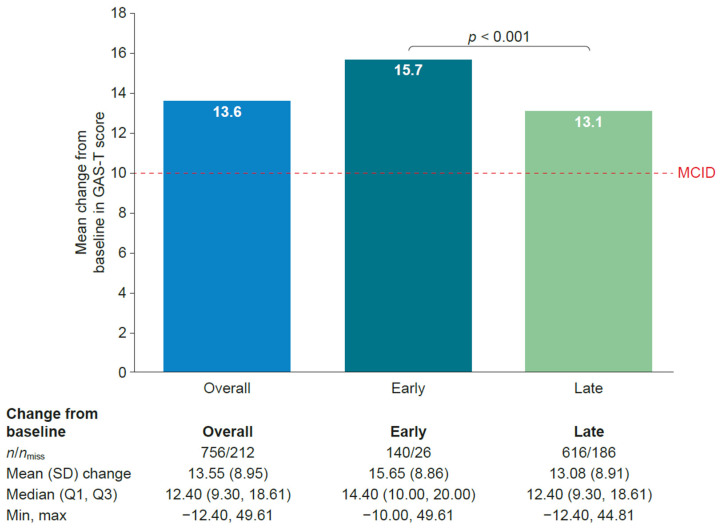
Change in unweighted GAS-T score from baseline to 12 (±2) weeks. The early treatment cohort consisted of patients who had BoNT-A treatment < 1 year post-stroke. The late treatment cohort consisted of patients who had BoNT-A treatment ≥ 1 year post-stroke. BoNT-A, botulinum toxin A; GAS-T, total goal attainment scaling; max, maximum; MCID, minimally clinically important difference; min, minimum; *n*, number of patients with a non-missing value; *n*_miss_, number of patients with a missing value; Q, quartile; and SD, standard deviation.

Mean total GAS (GAS-T) score at week 12 (±2) weeks improved from baseline in both cohorts. Patients in the early treatment cohort showed a statistically significantly greater improvement in mean GAS-T score than those in the late treatment cohort (*p* < 0.001). The mean (standard deviation) change from baseline in GAS-T score to 12 (±2) weeks was 15.7 (8.9) in the early treatment cohort and 13.1 (8.9) in the late treatment cohort. The results are shown in Figure 2.

### 2.3. Secondary Endpoints

Evaluated secondary endpoints were the number of patients with a GAS-T score ≥ 50 at 12 (±2) weeks and the change in MAS score by study from baseline to 12 (±2) weeks per treated muscle group.

#### 2.3.1. Functional Endpoint

The proportion of patients with a GAS-T score of ≥50 was greater in the early treatment cohort than in the late treatment cohort, with respective values of 63.9% and 47.4% at 12 (±2) weeks (*p* < 0.001), indicating that more patients in the early treatment cohort achieved their goals as expected than in the late treatment cohort.

#### 2.3.2. Muscle Tone

**Figure 3:**
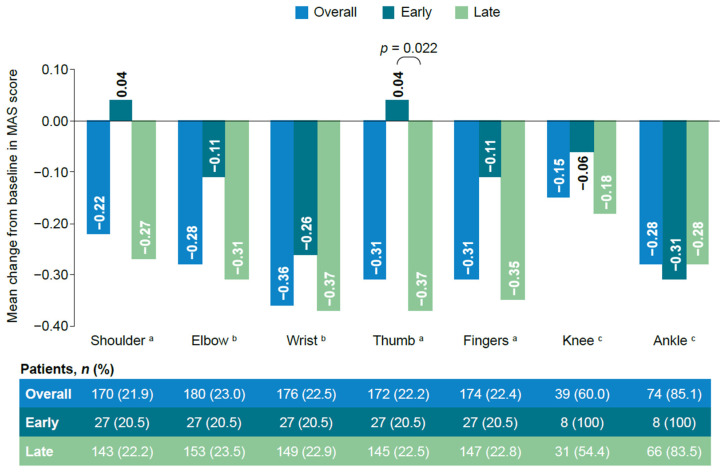
Change in MAS score from baseline to 12 (±2) weeks. ^a^ From study 1. ^b^ From studies 1 and 4. ^c^ From studies 2 and 3. MAS, Modified Ashworth Scale.

MAS scores generally decreased from baseline to week 12 in both cohorts (the lower the score, the greater the improvement). The changes were not statistically significantly different between the early versus late treatment cohorts for any of the muscle groups, with the exception of the thumb (Figure 3).

### 2.4. Exploratory Endpoints

The proportion of patients with a Physician’s Global Assessment (PGA) rating of “Much better” at 12 (±2) weeks was higher in the early treatment cohort versus the late treatment cohort (16.3% vs. 5.2%, *p* < 0.001), summarized in Table 2.

When stratified by prior receipt of BoNT-A therapy, the mean change in the GAS-T score from baseline to 12 weeks was similarly seen to be significantly higher in the early treatment cohort compared with the late treatment cohort for patients who were BoNT-A-naïve (*p* = 0.018), but not for those who had received prior BoNT-A therapy (Supplementary Table S1). A significantly higher proportion of patients in the early versus late treatment cohort had a GAS-T score ≥ 50 at 12 weeks for patients who were BoNT-A-naïve (*p* < 0.001), but not for those who had received prior BoNT-A therapy (Supplementary Table S1).

### 2.5. Safety

Safety data were available from one study (study 2), a real-world, prospective, longitudinal study evaluating the effectiveness of abobotulinumtoxinA (aboBoNT-A) for treating lower limb spasticity in adults (Supplementary Table S2). A higher proportion of patients in the early treatment cohort experienced treatment-emergent adverse events compared with the late treatment cohort (37.9% vs. 11.0%). The majority of adverse events reported were single events; no trends were observed. One occurrence of injection site hematoma in the early treatment cohort was considered related to study treatment. Four patients in the late treatment cohort reported serious adverse events, none of which were fatal, and all were considered unrelated to study treatment.

## 3. Discussion

Overall, this study suggested that early treatment of PSS with BoNT-A, within 1 year of stroke, had a positive impact on patients’ ability to achieve their treatment goals. GAS is a patient-centered measurement tool used to quantify progress toward specific, individualized goals [12,13]. The patient and clinician collaboratively define the goals, and GAS can capture functional changes that matter most to the individual patient, providing a meaningful assessment of progress that traditional scales, such as those focused solely on muscle tone (e.g., MAS), might miss [14]. The findings from the present study may be useful for planning the rehabilitation of stroke patients. GAS is particularly useful in rehabilitative and patient-focused care settings where the primary aim is to improve daily function and quality of life rather than just a single physiological measure such as muscle tone [15].

BoNT-A treatment was found to benefit patients in both the early and the late treatment cohorts in terms of functional improvement as measured by GAS and muscle stiffness. The change in GAS-T scores was clinically relevant in both cohorts, where the minimal clinically important difference was reported to be approximately 10 [16]. However, patients in the early treatment cohort showed statistically significantly greater improvements in GAS-T scores compared with those in the late treatment cohort from baseline to 12 (±2 weeks). When looking at GAS outcomes by previous BoNT-A therapy, patients who were BoNT-A-naïve showed similar findings to the main analysis; patients who had received prior BoNT-A therapy showed a similar pattern, but the differences were not statistically significant. This may be due to the small population in the early treatment cohort who had received prior BoNT-A therapy and needs to be confirmed in a larger population. Patients in both cohorts generally showed a reduction in MAS scores from baseline to 12 (±2) weeks, although there was no strong evidence to suggest any difference between the early versus late treatment cohorts, with the exception of the thumb. No unexpected safety concerns were reported. Although the frequency of treatment-emergent adverse events was higher in the early treatment cohort, the events reported were generally single events with no trends observed. Only one event was considered treatment-related and was in line with the known safety profile.

These data are consistent with previous studies, early-BIRD [17], ONTIME [18], and REFLEX [19]. The early-BIRD study evaluated the real-world effectiveness of aboBoNT-A on the evolution of spasticity in 303 patients with upper limb PSS according to the time from stroke to the start of BoNT-A treatment. Both the early-BIRD study and our study found that early treatment did not lead to statistically significant improvements in MAS, although in early-BIRD, reductions in MAS scores descriptively favored the early treatment group (within 6 months) versus the late treatment group [17]. A longitudinal cohort study evaluating the effects of BoNT/A in a real-world population of treatment-naïve patients with PSS who received injections within 12 months of stroke onset showed a significant reduction in muscle tone at both 4 and 12 weeks post-injection, particularly in patients treated within 90 days of stroke [20]. A secondary analysis of the data suggested that early BoNT-A treatment was also associated with enhanced reduction in spasticity and improved motor recovery [11].

In addition, in our study, patients in the early treatment group did better in achieving their treatment goals than those in the late treatment group. This suggests that while BoNT-A relaxes muscles regardless of when it is given, early treatment of PSS with BoNT-A can have a positive impact on patients’ ability to achieve treatment goals. In the ONTIME randomized controlled trial, patients with ULS received a fixed dose (500 U) of aboBoNT-A or placebo 2–12 weeks after stroke. Overall, 39.3% of patients receiving aboBoNT-A did not require re-injection for ≥28 weeks compared with 14.3% in the placebo group. The authors concluded that their study suggested an optimal time for PSS management, although they did not compare different time points for PSS treatment with BoNT-A [18]. The phase 3 REFLEX study evaluated the impact of early intervention with onabotulinumtoxinA (onaBoNT-A) treatment in adult patients with post-stroke lower limb spasticity. Earlier initiation of onaBoNT-A (≤24 months vs. >24 months since stroke) provided benefit to patients with some improvements in muscle tone and global functioning as measured by the physician global response scale and goal attainment; the authors concluded that early intervention may lead to improved patient outcomes [19]. In our study, PGA-based assessments also favored the early treatment cohort versus the late treatment cohort.

Challenges inherent to the PSS pathway may delay BoNT-A treatment with possible detrimental effects for patients on their rehabilitation outcomes and motivation to engage and persist with treatment. Recent studies have focused on ensuring that patients have access to appropriate and timely treatment for spasticity following stroke; for example, the ongoing EPITOME study is aiming to develop and implement a form of systematic monitoring of patients who have had a stroke to help with the detection of PSS in routine clinical practice [21]. By closely monitoring patients’ symptoms following a stroke, PSS can be detected earlier, leading to a swifter referral.

Early BoNT-A treatment following stroke may have a positive impact on patients’ ability to achieve their treatment goals and may support their rehabilitation outcomes. By setting and measuring patient-identified goals, patients may be empowered in their recovery program, through psychological engagement and motivation, to persist with therapy [22]. Furthermore, BoNT-A treatment post-stroke may have a disease-modifying effect, contributing to continued neuroplasticity in the early stages of recovery and potentially reducing neurological changes and related complications, leading to increased disability [17,18,23,24,25,26,27].

The strengths of this retrospective study include the use of a large data set with wide geographical coverage. The results suggest that early BoNT-A treatment of spasticity (i.e., initiated within 1 year of stroke) may lead to better goal attainment by patients compared with those who receive their first treatment with BoNT-A later after their diagnosis with spasticity. Goal attainment outcome measures may be more meaningful to patients than endpoints that focus on muscle tone alone, such as MAS, which may not translate into functional benefit.

A limitation of this study was that the source studies were conducted for reasons other than to evaluate early versus late treatment impact. Therefore, differences in the original trial study designs, such as sample sizes and geography, may bias the results of this analysis. In terms of geography, there was a greater proportion of patients from Russia in the early treatment cohort compared with other regions, and there was a greater proportion of patients from Europe in the late treatment cohort compared with other regions. Also, it is not known if patients in the early treatment cohort had better access to care than those in the late treatment cohort or if they were treated in healthcare systems with more structured post-stroke pathways. Some of the patients in each group had previously received BoNT injections; however, the response to previous treatment was not captured, and therefore, the potential impact on the interpretation of the study results is unknown.

## 4. Conclusions

Early treatment of PSS with BoNT-A, within 1 year of stroke, has a positive impact on patients’ ability to achieve their desired treatment goals. Further studies are required to investigate how soon after receiving early BoNT-A treatment patients achieve their goals.

## 5. Materials and Methods

### 5.1. Study Design and Data Source

This was a retrospective cohort study using secondary and anonymized data from patients with PSS who received treatment with BoNT-A. Data were pooled from five company-sponsored studies, summarized in Table 3 (study 1, NCT02454803/ULIS III; study 2, NCT04050527/AboLiSh; study 3, NCT02020980/RELIEF; study 4, NCT01682148; and study 5, A-92-52120-168).

The source studies collected data on time since stroke diagnosis to BoNT-A treatment, functional outcomes of interest, as well as other clinical outcomes at similar time points (12 and 24 [±2] weeks following the enrollment visit).

### 5.2. Patients

Eligible patients were adults (aged ≥ 18 years) with PSS who received at least one injection of BoNT-A and for whom the decision to administer BoNT-A treatment for PSS had been made before study enrollment. Exclusion criteria included patients with no GAS measurement at baseline or no valid GAS measurement post-baseline (at 12 and 24 [±2] weeks). Baseline was defined as the inclusion visit or a previous visit at which an injection was administered or an evaluation cycle was initiated. Patients meeting all prespecified inclusion and exclusion criteria were categorized into two cohorts (early or late) according to the duration of time between their stroke and BoNT-A treatment. The early cohort consisted of patients who had BoNT-A treatment within the first year (<1 year) after their recorded stroke event. The late cohort consisted of patients who had BoNT-A treatment 1 year or later (≥1 year) after their recorded stroke event.

### 5.3. Endpoints

The primary endpoint was the change in GAS-T score from baseline to 12 (±2) weeks. GAS scores are an individualized outcome measure involving goal selection and scoring to assess whether the predefined goals were met. Scores were combined to give an aggregated GAS-T score [12] and described as a continuous variable. GAS scores were unweighted.

The secondary endpoints included the number of patients with a GAS-T score ≥ 50 at 12 (±2) weeks and the change in MAS score from baseline to 12 (±2) weeks per treated muscle group (shoulder, elbow, wrist, thumb, fingers, ankle, and knee). MAS was used to measure muscle tone using a scale from 0 (no increase in muscle tone) to 4 (affected part[s] rigid in flexion or extension). The feasibility of analyzing the data with MAS as a categorical variable was assessed; however, because the different studies collected MAS scores from different joints, there were large amounts of missing data, and analysis was not possible.

Exploratory analyses included the number of patients within each PGA category at 12 (±2) weeks. PGA measures the clinicians’ perception of patient outcomes using a generic rating scale to measure response to treatment (much better, a bit better, same, worse, much worse). GAS scores were also evaluated by prior BoNT-A therapy status (prior or no prior BoNT-A therapy). Although not part of the prespecified endpoints in this analysis, safety data were available from study 2.

For each endpoint, a comparison was made between the early versus late treatment cohorts to explore the impact of BoNT-A timing post-stroke on treatment outcomes.

### 5.4. Sample Size and Statistical Analysis

A feasibility assessment of the availability of GAS data in the pooled study population (*N* = 1735) showed that 968 patients had GAS assessment data at both baseline and at 12 (±2) weeks and were suitable for the assessment of the primary endpoint. The statistical analyses were performed using the R programming language version 4.2.2 or higher, in accordance with the International Council for Harmonisation of Technical Requirements for Pharmaceuticals for Human Use (ICH) E9 guideline and based on the pooled data from the individual studies, unless otherwise stated. Descriptive analyses were performed to evaluate all primary, secondary, and exploratory endpoints for the early and late treatment cohorts. Baseline patient and clinical characteristics were described; continuous variables were summarized by n available, mean, SD, median, first and third quartiles, minimum and maximum, and categorical variables were summarized by overall n available, frequency, and percentage for each category. An assessment of missing data was presented as the number (percentage) of missing values (missing, *n* [%]) for each assessed endpoint and associated variables.

The impact of BoNT-A treatment timing on treatment outcome was evaluated in terms of goal attainment and analyzed in two ways by comparing the change from baseline in the GAS-T score to 12 (±2) weeks for patients in the early versus late treatment cohorts and the proportion of patients with a GAS-T score ≥ 50 at 12 (±2) weeks in the early versus late treatment cohorts. MAS was evaluated in terms of MAS score change from baseline to 12 (±2) weeks for any of the muscle groups and compared for the early versus late treatment cohorts. The impact of BoNT-A treatment timing on the exploratory endpoint was compared for the early versus late treatment cohorts by evaluating the number of patients within each PGA category at 12 (±2) weeks. Statistically significant differences between the two groups for each endpoint variable were assessed using the Wilcoxon rank sum test for continuous variables due to violations of normal distributions through the Q–Q plots and the Chi-squared test for categorical variables. All statistical tests were two-sided and performed at the 5% level of significance.

## Figures and Tables

**Table 1:** Baseline patient characteristics by early and late BoNT-A treatment.

Characteristic	Overall (*n* = 968)	Early Treatment (<1 year) (*n* = 166)	Late Treatment (≥1 year) (*n* = 802)	*p* Value
Study, *n* (%)				
1. NCT02454803	776 (80.2)	132 (80.0)	644 (80.3)	Not calculated
2. NCT04050527	147 (15.2)	29 (17.5)	118 (14.7)	
3. NCT02020980	28 (2.9)	0 (0)	28 (34.9)	
4. NCT01682148	6 (0.6)	0 (0)	6 (0.7)	
5. Study 5	11 (1.1)	5 (3.0)	6 (0.7)	
Male sex, *n* (%)	565 (58.4)	109 (65.7)	456 (56.9)	0.045
Age group, years, *n* (%)				
18–34	96 (9.9)	15 (9.0)	81 (10.1)	>0.9
35–44	122 (12.6)	19 (11.4)	103 (12.8)	
45–49	97 (10.0)	18 (10.8)	79 (9.9)	
50–54	137 (14.2)	27 (16.3)	110 (13.7)	
55–59	148 (15.3)	24 (14.5)	124 (15.5)	
60–64	99 (10.2)	18 (10.8)	81 (10.1)	
65–69	119 (12.3)	18 (10.8)	101 (12.6)	
≥70	150 (15.5)	27 (16.3)	123 (15.3)	
Region, *n* (%)				
America	150 (15.5)	20 (12.0)	130 (16.2)	<0.001
Asia or Australia	154 (15.9)	32 (19.3)	122 (15.2)	
Europe	460 (47.5)	52 (31.3)	408 (50.9)	
Russia	204 (21.1)	62 (37.3)	142 (17.7)	
Location of spasticity, *n* (%)				
Upper limb only	793 (81.9)	137 (82.5)	656 (81.8)	0.2
Lower limb only	26 (2.7)	1 (0.6)	25 (3.1)	
Upper and lower limb	149 (15.4)	28 (16.9)	121 (15.1)	
Time from spasticity diagnosis to BoNT-A treatment, years ^a^				
*n* (%)	676 (69.8)	125 (75.3)	551 (68.7)	<0.001
Mean (SD)	5.91 (7.39)	0.50 (1.28)	7.14 (7.64)	
Median (Q1, Q3)	3.49 (1.16, 7.63)	0.36 (0.20, 0.59)	4.79 (2.06, 8.63)	
Min, max	0.01, 52.02	0.01, 13.93	0.12, 52.02	
Time from stroke to BoNT-A treatment, years				
Mean (SD)	6.67 (8.05)	0.50 (0.26)	7.95 (8.29)	<0.001
Median (Q1, Q3)	4.00 (1.46, 8.57)	0.47 (0.27, 0.73)	5.42 (2.61, 9.94)	
Min, max	0.02, 64.65	0.02, 0.99	1.01, 64.65	
Previously treated with BoNT-A for spasticity, *n* (%)				
Yes	667 (68.9)	48 (28.9)	619 (77.2)	<0.001
No	301 (31.1)	118 (71.1)	183 (22.8)	
Baseline unweighted GAS scores				
Mean (SD)	36.87 (3.62)	36.90 (3.53)	36.86 (3.64)	0.900
Median (Q1, Q3)	37.60 (36.31, 40.00)	37.60 (36.31, 40.00)	37.60 (36.31, 40.00)	
Min, max	22.61, 40.00	24.83, 40.00	22.61, 40.00	

Data were from all pooled studies except where indicated. ^a^ From all studies except study 4. BoNT-A, botulinum toxin A; GAS, goal attainment scaling; max, maximum; min, minimum; Q, quartile; and SD, standard deviation.

**Table 2:** Physician’s Global Assessment at 12 (±2) weeks ^a^.

Rating	Overall (*n* = 968)	Early Treatment (<1 year) (*n* = 166)	Late Treatment (≥1 year) (*n* = 802)	*p* Value
Total patients	237 (24.5)	53 (31.9)	184 (22.9)	
Much better	69 (7.1)	27 (16.3)	42 (5.2)	<0.001
A bit better	127 (13.1)	20 (12.0)	107 (13.3)	
Same	39 (4.0)	5 (3.0)	34 (4.2)	
Worse	2 (0.2)	1 (0.6)	1 (0.1)	
Much worse	0 (0)	0 (0)	0 (0)	

^a^ From studies 1, 2, and 5. Data are presented as *n* (%).

**Table 3:** A description of the five studies from which the data were pooled.

Study	Study Design	Objective	Population
1. NCT02454803 ULIS III [28]	International, multicenter, observational, prospective, longitudinal cohort study conducted at 60 centers in countries with a Marketing Authorization for at least one BoNT-A preparation approved for ULS treatment	To assess the impact of integrated ULS management (including BoNT-A injections) on patient-centered goal attainment in patients	Patients aged ≥ 18 years over a period of 2 years
2. NCT04050527 AboLiSh [29]	International, multicenter, observational, prospective, longitudinal study conducted at 51 centers in 9 countries in Europe, the Americas, Australia, and Russia	To assess the effectiveness (longitudinal attainment of person-centered and function-related goals) of aboBoNT-A injections in patients	Patients aged ≥ 18 years with LLS over a period of 16 months
3. NCT02020980 RELIEF	National, prospective, multicenter, post-marketing observational study conducted at 23 centers in Spain	To demonstrate the effect of intramuscular BoNT-A injections in relieving pain consequent to post-stroke LLS as per routine clinical practice	Patients aged ≥ 18 years with post-stroke LLS
4. NCT01682148 [30]	A randomized, evaluator-blinded, comparative, parallel-group, multicenter study conducted in 4 countries (Denmark, Finland, Norway, and Sweden)	To compare aboBoNT-A treatment results in the elbow joint 4 weeks post-treatment using MAS with the following two techniques: current clinical practice (300 U/mL) versus neuromuscular junction-targeted injection (100 U/mL)	Patients aged ≥ 18 years with ULS with an elbow flexor muscle spasticity position pattern type 1, 3, or 4 post-stroke or traumatic brain injury
5. A-92-52120-168	A post-marketing, national, multicenter, prospective, observational, longitudinal, open, and single-cohort study conducted at 6 centers in Spain	To evaluate post-stroke ULS patterns in patients before and after treatment with BoNT-A, according to the determined postural and movement patterns	Patients aged ≥ 18 years with post-stroke ULS

aboBoNT-A, abobotulinumtoxinA; BoNT-A, botulinum toxin A; LLS, lower limb spasticity; MAS, Modified Ashworth Scale; and ULS, upper limb spasticity.

## Data Availability

Qualified researchers with a valid research question may request anonymized patient-level data by contacting an Ipsen representative. Further information on Ipsen’s Data Sharing policy is available here (Clinical Data Transparency–Global, https://www.ipsen.com/science/clinical-trials/clinical-data-transparency/ accessed on 14 January 2026).

## Supplementary Materials

The following supporting information can be downloaded at: https://www.mdpi.com/article/10.3390/toxins18020068/s1. Table S1: GAS outcomes by prior BoNT-A therapy status; Table S2: Summary of safety data from study 2.

## Abbreviations

The following abbreviations are used in this manuscript:

aboBoNT-A	AbobotulinumtoxinA
AE	Adverse event
BoNT-A	Botulinum toxin A
GAS	Goal attainment scaling
GAS-T	Total Goal Attainment Scaling
ICH	International Council for Harmonisation of Technical Requirements for Pharmaceuticals for Human Use
LLS	Lower limb spasticity
MAS	Modified Ashworth Scale
MCID	Minimal clinically important difference
PGA	Physician’s Global Assessment
PSS	Post-stroke spasticity
TEAE	Treatment-emergent adverse event
ULS	Upper limb spasticity
