# Supplementary material for: Impact of Early Versus Late Treatment with Botulinum Toxin A on Goal Attainment in Post-Stroke Spasticity: A Retrospective Cohort Study
Source: Toxins (Basel). 2026 Jan 27;18(2):68. doi: 10.3390/toxins18020068 (PMC12945037; doi:10.3390/toxins18020068)
Supplement: Supplementary file 1 [file toxins-18-00068-s001.zip › toxins-4010620-supp-update.pdf]

Supplementary Materials: Impact of Early Versus Late Treatment with Botulinum Toxin A on Goal Attainment in Post-Stroke Spasticity: A Retrospective Cohort Study

Atul Patel, Jinming Zhang, Simon Page, Sarah Harding, Mathieu Beneteau, Colin Navickas and Alberto Esquenazi

Supplementary Table S1. GAS outcomes by prior BoNT-A therapy status.

	**Prior BoNT-A Therapy**				**No Prior BoNT-A Therapy**			
	**Early Treatment (n = 48)**	**Late Treatment (n = 619)**	**95% CI**	***p* Value**	**Early Treatment (n = 118)**	**Late Treatment (n = 183)**	**95% CI**	***p* Value**
**Baseline unweighted GAS scores**								
Mean (SD)	36.73 (3.91)	36.84 (3.64)	−1.3, 1.1	0.8	36.98 (3.38)	36.94 (3.67)	−0.77, 0.85	0.5
Median	37.60	37.60			37.60	37.60		
(Q1, Q3)	(36.31, 40.00)	(36.31, 40.00)			(37.60, 40.00)	(37.60, 40.00)		
Min, max	24.83, 40.00	22.61, 40.00			25.19, 40.00	22.61, 40.00		
**Change in unweighted GAS-T scores from baseline to 12 (± 2) weeks**								
n (%)/ n_{miss} (%)	37 (77.1)/ 11 (22.9)	484 (78.2)/ 135 (21.8)			103 (87.3)/ 15 (12.7)	132 (72.1)/ 51 (27.9)		
Mean (SD)	14.08 (11.79)	12.95 (8.89)	−2.9, 5.1	0.7	16.21 (7.53)	13.56 (9.00)	0.52, 4.8	0.018
Median	11.41	10.71			15.50	12.40		
(Q1, Q3)	(9.30, 15.50)	(9.13, 18.61)			(12.40, 20.00)	(9.30, 18.61)		
Min, max	−10.00, 49.61	−10.00, 44.81			0.00, 37.21	−12.40, 37.21		
GAS-T scores ≥50 at 12 (± 2) weeks, n (%)								
Yes	25 (52.1)	293 (47.3)		0.7	81 (68.6)	87 (47.5)		<0.001
No	12 (25.0)	191 (30.9)			22 (18.6)	45 (24.6)		
Missing	11 (22.9)	135 (21.8)			15 (12.7)	51 (27.9)		

BoNT-A, botulinum toxin A; CI, confidence interval; GAS, goal attainment scaling; GAS-T, total GAS; max, maximum; min, minimum; n, number of patients with a non-missing value; n_{miss}, number of patients with a missing value; Q, quartile; SD, standard deviation.

Supplementary Table S2. Summary of safety data from study 2.

	**Overall (*n* = 147)**	**Early Treatment (<1 year) (*n* = 29)**	**Late Treatment (≥1 year) (*n* = 118)**
**Any TEAE, *n* (%)**	24 (16.3)	11 (37.9)	13 (11.0)
TEAEs per patient (only among those with an event)			
n (%)	24 (16.3)	11 (37.9)	13 (11.0)
Mean (SD)	1.79 (2.02)	1.00 (0)	2.46 (2.60)
Median (Q1, Q3)	1.00 (1.00, 1.25)	1.00 (1.00, 1.00)	1.00 (1.00, 2.00)
Min, max	1.00, 10.00	1.00, 1.00	1.00, 10.00
TEAEs per patient (among all patients)			
n (%)	147 (100)	29 (100)	118 (100)
Mean (SD)	0.29 (1.04)	0.38 (0.49)	0.27 (1.14)
Median (Q1, Q3)	0 (0, 0)	0 (0, 1.00)	0 (0, 0)
Min, max	0, 10.00	0, 1.00	0, 10.00
Any severe AE, *n* (%)	3 (2.0)	0 (0)	3 (2.5)
Any AE considered related to study drug, *n* (%)	1 (0.7)	1 (3.4)	0 (0)
Any serious AE, *n* (%)	4 (2.7)	0 (0)	4 (3.4)
Any AE considered related to death, *n* (%)	0 (0)	0 (0)	0 (0)

AE, adverse event; max, maximum; min, minimum; Q, quartile; SD, standard deviation; TEAE, treatment-emergent adverse event.
